# Deep Learning Assisted Buildings Energy Consumption Profiling Using Smart Meter Data

**DOI:** 10.3390/s20030873

**Published:** 2020-02-06

**Authors:** Amin Ullah, Kilichbek Haydarov, Ijaz Ul Haq, Khan Muhammad, Seungmin Rho, Miyoung Lee, Sung Wook Baik

**Affiliations:** 1 Intelligent Media Laboratory, Digital Contents Research Institute, Sejong University, Seoul 143-747, Korea; qamin3797@gmail.com (A.U.); kilichbek.haydarov@gmail.com (K.H.); ijazulhaq@sju.ac.kr (I.U.H.); miylee@sejong.ac.kr (M.L.); 2 Department of Software, Sejong University, Seoul 143-747, Korea; khan.muhammad@ieee.org (K.M.); smrho@sejong.ac.kr (S.R.)

**Keywords:** artificial intelligence, big data, clustering, energy consumption prediction, buildings energy management, smart sensing

## Abstract

The exponential growth in population and their overall reliance on the usage of electrical and electronic devices have increased the demand for energy production. It needs precise energy management systems that can forecast the usage of the consumers for future policymaking. Embedded smart sensors attached to electricity meters and home appliances enable power suppliers to effectively analyze the energy usage to generate and distribute electricity into residential areas based on their level of energy consumption. Therefore, this paper proposes a clustering-based analysis of energy consumption to categorize the consumers’ electricity usage into different levels. First, a deep autoencoder that transfers the low-dimensional energy consumption data to high-level representations was trained. Second, the high-level representations were fed into an adaptive self-organizing map (SOM) clustering algorithm. Afterward, the levels of electricity energy consumption were established by conducting the statistical analysis on the obtained clustered data. Finally, the results were visualized in graphs and calendar views, and the predicted levels of energy consumption were plotted over the city map, providing a compact overview to the providers for energy utilization analysis.

## 1. Introduction

In recent years, the global demand for energy has propelled due to population growth, the utilization of electrical and electronic devices, economic development, and dramatic changes in climate. The residential sector is regarded as one of the major consumers of energy [1]. According to the United Nations Environment Program [2], buildings consume approximately 40% of global energy. This fact brings huge challenges to the power-supply side and energy management systems. Nevertheless, at the same time, it stimulates data scientists to come up with new methods for energy consumption predictions, which contribute to optimal energy usage and a more balanced distribution from the power supply side [3]. Smart sensing technologies collect data from multiple sources such as room temperature, humidity, wind speed, heating load, cooling load, and the energy consumption of all home appliances. This multivariant data provide an analysis platform for the data scientist to utilize it for forecasting and effective energy consumption management.

Energy consumption is dependent on many factors such as the infrastructure, energy prices, and weather conditions. Therefore, it is very difficult to forecast the overall energy demand of residential buildings by simulating each building performance with conventional systems [4]. Alternatively, machine learning and data mining techniques can be utilized, which exploit knowledge from historical data and can provide enough decision-making predictions, the basis for designing new power distribution configurations for residential areas [5,6].

In the past decade, several machine learning techniques including supervised and unsupervised approaches have been utilized for energy consumption level predictions [7]. Among the unsupervised learning techniques, clustering is considered as one of the most frequently applied techniques in data mining and machine learning. Clustering involves partitioning objects with similar patterns under observation into different groups. A vast number of works on clustering electricity usage patterns have been presented by researchers. For instance, Kim et al. [8] generated a typical load profile from data measured with automatic meter reading systems, then performed cluster analysis using three clustering algorithms, specifically, the hierarchical, k-means, and fuzzy c-means algorithms. Hernandez et al. [9] classified daily load curves in industrial parks, which can be regarded as microgrids from the energy network perspective using SOM, then exploited k-means to obtain a number of clusters. Ford and Siraj [10] demonstrated the possibility of applying disaggregation techniques on smart meter data via fuzzy c-means clustering. A similar work is attributed to Rhodes et al. [11] where they utilized the k-means algorithm to group residential houses with similar hourly electricity use profiles. Ramos et al. [12] proposed a method to characterize electricity medium voltage consumers by using several clustering algorithms. In order to choose the best one among the typical load profiles, they measured the performance of the clustering algorithms in terms of eight clustering validity indices. To deal with scalability and computational complexity of the power consumption profiling process, Al-Jarrah et al. [13] proposed the multi-layered clustering method for power consumption profiling. First, they acquired local power consumption profiles using k-means, considering clusters with a low number of patterns as abnormal power consumption behavior. In the second stage, a global power consumption profile was derived from the local ones. Furthermore, Cai et al. [14] applied an improved k-means algorithm with particle swarm optimization (PSO) to open residential buildings dataset to divide their electricity consumption in an entire region into different levels. To extract the daily electricity consumption behavior of a household, Nordahl et al. [15] used the centroids of the generated clusters by k-medoids. Park and Son [16] developed a methodology in which one-dimensional time series smart meter data were reshaped to two-dimensional arrays called load profile images. After performing image processing techniques on those images, they derived the class load image profiles via clustering algorithms. Rasanen et al. [17] partitioned customers into electricity user groups based on similar electricity usage behavior with the SOM, k-means, and hierarchical clustering algorithms. Similarly, to group electricity consumption profiles, Wen et al. [18] investigated a shape-based clustering method.

The discussed literature reveals several limitations of the employed techniques from various perspectives of energy consumption prediction. The aforementioned literature lacks focus on capturing the recognizable patterns in building smart sensing data, which has a limited number of features. These features can be represented in low dimensional feature space and may affect the overall performance in data analytic tasks. The majority of the existing techniques enquire about the number of clusters to differentiate among distinct categories of data. In addition, the presentation of the energy consumption for data analysts and common individuals is a common problem that has not been tackled effectively in the existing literature. Therefore, aiming at the mentioned problems in energy predictions for households, this paper presents a novel framework with the following main contributions:

(1) The energy consumption data acquired from residential building smart sensors are of very low dimensions, and finding recognizable patterns in such data is very difficult, which affects the performance of electrical energy consumption analysis. To address this issue, a deep autoencoder, which effectively learns and converts the tiny pattern representations in low dimensional data into high-level representations, is proposed.

(2) The mainstream clustering algorithms require an input parameter to divide data into multiple clusters. In this paper, an adaptive clustering algorithm known as self-organizing maps (SOMs) was utilized to efficiently divide the high dimensional data achieved from the pre-trained deep autoencoder into multiple clusters.

(3) After the division of data into multiple clusters, statistical analysis was performed on the clustered data to know which buildings had a higher-, mid-, and low- level of energy consumption.

(4) The energy providers are not always data analysists who can understand the processed information easily. Therefore, after clustering and finding the levels of energy consumption, the information was visualized on the map of the city from where the data were gathered. This helps the proposed framework to precisely present the electricity consumption data of different areas of the city to the providers.

The rest of the paper is organized as follows. All technical details of the proposed framework are discussed in Section 2. The experimental evaluation and discussion on the results are given in Section 3. Section 4 summarizes the key findings of this article and our recommendations for future research.

## 2. Proposed Method

**Figure 1:**
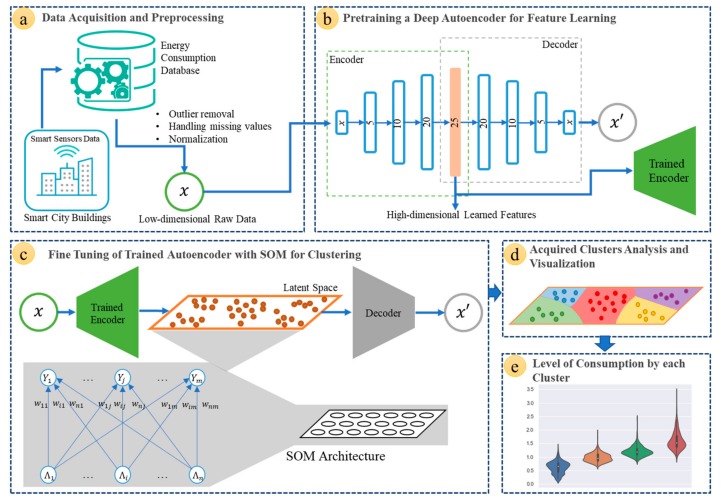
Framework of the proposed building energy consumption level prediction: (**a**) Data are obtained from the buildings’ smart sensing devices and preprocessed for removing outliers and normalization, (**b**) a deep autoencoder is pre-trained to convert low dimensional sensors data into higher-level representations, (**c**) the pre-trained autoencoder is trained along with the SOM clustering layer, (**d**) visualization of the obtained clusters, (**e**) finding the energy consumption level of each cluster data.

In this section, the core steps of the proposed methodology are discussed in detail. Figure 1 demonstrates the overall dataflow of the proposed framework, which is divided into five steps. First, data are acquired from the smart sensors of the buildings’ electricity meters. Next, the data goes through the pre-processing stage. Afterward, the deep autoencoder is pre-trained. At the fourth stage, the pre-trained autoencoder is trained jointly with a clustering layer through which a number of clusters are obtained. Finally, each cluster is defined with the level of energy consumption.

### 2.1. Datasets

In this study, the experimental results were conducted over two benchmark datasets. These datasets are fundamentally different from each other in a sense that the first dataset contains energy consumption smart sensors data from residential buildings in a city whereas the second one represents the data of a single house. This means that the proposed system was assessed from different viewpoints: analyzing the whole city and focusing on the single house. First, the proposed technique was examined on the dataset of the monthly energy consumption of Gainesville, located in Alachua County (Florida, USA), which is available via the Open Energy Information website [19]. The data contained the monthly electricity usage of 29,393 residential buildings measured in kWh over the period of five years from 2006 to 2010. During data inspection, some inconsistent samples and outliers were detected. Therefore, they were effectively removed from the dataset because their percentage was insignificant. Next, a single feature defined as kWh per area was used for the experiment. For this purpose, initially, a gross floor area of each building was extracted from the residential buildings’ characteristics data, and then the electricity energy consumption of each house was divided by their gross area. Finally, this single feature was passed into a proposed deep autoencoder.

The second dataset is available on the UCI Repository [20]. It holds 2,075,259 measurements of electric power consumption collected from December 2006 to November 2010 in a house situated in Sceaux, near Paris, France. Some missing values were present in the data, which comprised around 1.25% of the whole data. Pre-processing steps were applied, and all samples were normalized. The original data were recorded with a sampling rate of one minute. Therefore, the minutely data were resampled to a daily one to obtain the level of electricity energy consumption on a daily basis.

### 2.2. Smart Sensors Data Acquisition and Pre-processing

**Figure 2:**
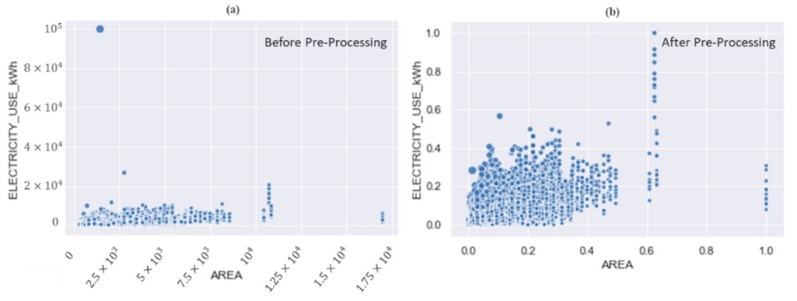
A comparison of the smart sensor data distributions (**a**) before and (**b**) after the pre-processing step.

It is a common fact and proven from recent studies that the performance of trained artificial intelligence (AI) models depend upon the data. Therefore, if the data are accurate and well organized, it helps to precisely train any AI model. Furthermore, the real-world energy consumption data gathered from different kinds of sensing devices are stored in raw format, often incomplete, not well organized, and inconsistent. Therefore, in the proposed framework, the raw data first undergoes the data pre-processing stage, where the noise and outliers are removed. The data distributions before and after the pre-processing step is given in Figure 2 where it can be seen that one sample is an outlier and separated from the other data samples, which would make the training ineffective, therefore, all such samples were removed from the data. Furthermore, as data loss may be encountered during data transmission in networks, some values in the data might be absent. Therefore, in the proposed framework, the missing values in the data were handled by utilizing the mean imputation method [21]. The dataset used for the experiments had only 1.25% of missing values, which were very precisely handled by the mean imputation method. Finally, the min–max method was applied for the normalization of data [22], which maps the entire range of values from 0 to 1 in such a way that the minimum and maximum values become 0 and 1, respectively. The effect of normalization can be seen in Figure 2 where the data distributions are much better to divide into multiple clusters.

### 2.3. Deep Autoencoder for Feature Learning

Artificial neural networks (ANNs) have been extensively used in the past decade and have different variants that are used for various kinds of data analysis [23]. The autoencoders are one of the effective types of ANN, which are mostly used for compressing data from high to low dimension features or expending from low to high dimension features. It efficiently learns the patterns in data, encodes it, squeezes, or expand it, and reconstructs it to the original form by utilizing the ANN backpropagation technique to tune its parameters and reduce the error rate. Autoencoders have been investigated in research areas such as image super-resolution [24] and denoising [25,26]. Ribeiro et al. [27] used the reconstruction error of appearance and motion features with a combination of video frames from a convolutional autoencoder to detect anomalous behavior. In addition, Zhavoronkov et al. [28] applied a variational autoencoder to map the chemical structure into a latent vector in their approach on the generative molecular design.

The typical autoencoder consists of two parts [29], the first part is called an encoder $f_{W}:x\to z$, where the model learns how to squeeze or expand the input dimensions and convert it into a latent-space representation based on Equation (1).

(1) $$z=f\left(x\right)=\;\sigma \left(Wx+b\right)$$

where σ is an activation function that can be sigmoid, tanh, and ReLU, etc. In the proposed technique, the ReLU activation function was utilized. *W* is the trainable weights, *b* is the bias unit, and *x* is the input value. The second part of the autoencoder is a decoder $g_{W}:z\to x^{\prime }$, where the model learns how to convert the encoded data back to its original representation based on Equation (2).

(2) $$x^{\prime }=g\left(z\right)=\;\sigma \left(W^{\prime }z+b^{\prime }\right)$$

The encoder and decoder are symmetrical to each other and share an intermediate layer known as a bottleneck, where the compressed or the extended data lies. In the deep structure of autoencoders, the data from the previous layer are input *x* to the next layer; the processing formula for deep and simple autoencoders is the same. Considering the energy consumption level prediction problem, the acquired energy data from different smart sensors is not sufficient to train a precise AI model. Recent studies indicate that effective AI models need a huge amount of data of intermediate dimensions. To address the issue of less data, researchers have utilized different kinds of oversampling techniques. For high to intermediate dimensions, researchers have utilized principal component analysis (PCA) and autoencoders. However, the low to high dimensions problem is very rarely addressed by data scientists because in this case, it is very difficult to reproduce the exact form of original data. In the proposed technique, a deep autoencoder has been presented to learn the patterns in low dimensional features so that high-level representations can be extracted from the energy consumption data. This idea was adopted from the deconvolution process in the recent convolutional neural network models such as FlowNet and FlowNet2 where they applied 2D filters to reconstruct an image from low dimensional features. Therefore, the design of the proposed autoencoder is different from the conventional structure of autoencoders where it is utilized to seek a non-linear mapping between low-dimensional original features and higher-dimensional latent feature space. For instance, given an input $x\;\epsilon \;{\mathbb{R}}^{d}$, the encoder maps it to $z\in {\mathbb{R}}^{p}$, where *d* is the dimensions of *x* and *p* is the dimensions of *z*, such that *d* < *p*, resulting in high-dimensional features in latent space being extracted using four encoding layers. The first layer encodes x-dimensional feature vector to five neurons, followed by 10, 20, and 25 dimensions, respectively. The five neurons increase between the autoencoder layers was chosen because the first dataset only had two features and similarly four features in the second dataset. Therefore, a direct increase from two to 25 is inefficient and the model faces an overfitting problem if it is trained for many iterations. The proposed deep autoencoder has been trained from scratch for 1000 epochs to estimate the non-linear mapping function $f_{W}$. During this phase, the learnable weights are adjusted iteratively by minimizing the difference between the original input and its reconstruction via the ANN backpropagation technique. The trained deep autoencoder was utilized jointly with SOM for clustering household energy data.

### 2.4. Fine-Tuning Train Deep Autoencoder with SOM for Clustering

Transfer learning, which is also known as fine-tuning, is a very effective process of patterns finding in raw data by utilizing AI trained models for different tasks. For instance, the famous convolutional neural network models including AlexNet, GoogleNet, and VGGNet are first trained on a large scale image dataset, then utilized for different tasks like object detection, tracking, and salient information extraction etc. using a parameter fine-tuning process [30]. In the fine-tuning process, the parameters of the model are not randomly initialized, but are tuned from the values it is already trained in, which helps the cost function to easily fit the underlying problem without going into any overfitting or underfitting problem. The proposed problem is related to clustering, where the energy consumption data are being divided into multiple clusters. The existing clustering techniques are mostly based on statistical formulas and are very difficult to fine tune with the trained neural network models. Second, for these techniques such as k-means [31], Density-based spatial clustering of applications with noise (DBSCAN) [32], Ordering points to identify the clustering structure (OPTICS) [33], etc., direct features are needed to feed to these algorithms, not the raw data that needs to be processed first for patterns extraction. Furthermore, it is very complicated to fuse statistical methods with the AI trainable parameters for the clustering task. Therefore, in the proposed technique, a NN based clustering technique is utilized for clustering by adding its layers to the bottleneck of the pre-trained deep autoencoder.

**Figure 3:**
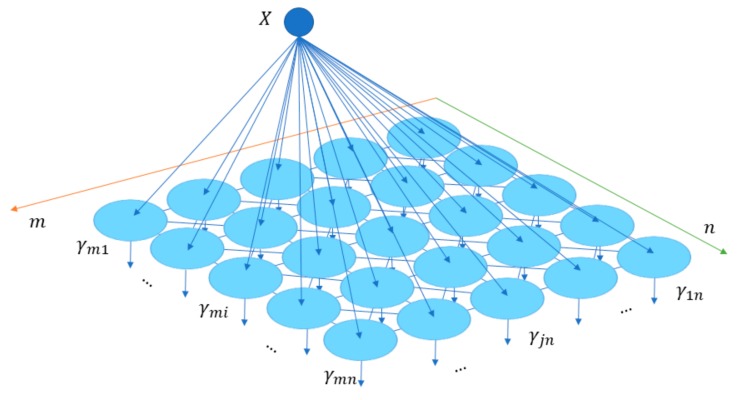
The internal structure of the SOM. A feature vector *X* is provided to the SOM map with the size of m × n, and the best matching unit is selected among the grid neurons $\gamma_{ij}\;\left(1\leq i\leq m\;and\;1\leq j\leq n\right).$ Afterward, the weight vectors of the winner unit and its neighboring units are updated. When the learning process is completed, each sample is assigned to its nearest node [35].

Given an estimate of the non-linear mapping function $f_{W}$, the deep autoencoder and clustering layer are jointly trained. The input for the cluster layer is learned latent space from the bottleneck of the pre-trained model. For clustering, the SOM was chosen, also known as Kohonen’s map [34]. The internal structure of the SOM is given in Figure 3. It consists of one- or two-dimensional grid of *N* processing units also called neurons. Each unit or neuron n is associated with a prototype vector $v_{n}=[v_{n1},\ldots ,\;v_{n\delta }$], where δ is the dimension of an input vector. The SOM stands out from other NN based techniques because it utilizes a neighborhood function to preserve the topological structure of the input space. Another property of the SOM is that it is based on competitive learning: the neurons whose weights vector match the input sample most closely according to some distance function are selected as the winner neurons *b* (Equation (3)). The weights of the winner neurons and its neighbors in the gird are updated toward the input sample with an adaptation coefficient $\alpha \left(t\right)$ according to an update rule in Equation (4). In this study, the neighboring units are determined by a Gaussian neighborhood function defined in Equation (5).

(3) $$\|x-v_{b}\|=\underset{n}{min}\left\{\left\|x-v_{n}\right\|\right\}$$

(4) $$v_{n}\left(t+1\right)=v_{n}\left(t\right)+\alpha \left(t\right)K\left(d\right)\left[x-v_{n}\left(t\right)\right]$$

(5) $$K\left(d\right)=\;e^{-{\left(\frac{d}{\lambda \left(t\right)}\right)}^{2}}$$

where *d* is the Manhattan distance between the winner neuron *b* and the other neuron *n* on the SOM map. The temperature parameter $\lambda \left(t\right)$ controls the radius of the neighborhood and shrinks linearly with time *t* as calculated in Equation (6).

(6) $$\lambda \left(t\right)=T_{max}-\frac{t}{N}\times \left(T_{max}-T_{min}\right),\;t=0,\;1,\;2,\ldots$$

where $T_{max}$ and $T_{min}$ correspond to the maximum and minimum temperatures, respectively. *N* is the maximum number of iterations for the input pattern *x*. Once the SOM network is trained, during the mapping process, when the testing sample is presented as an input, only the best matching unit is selected as a cluster label.

### 2.5. Level of Consumption by Each Cluster

**Figure 4:**
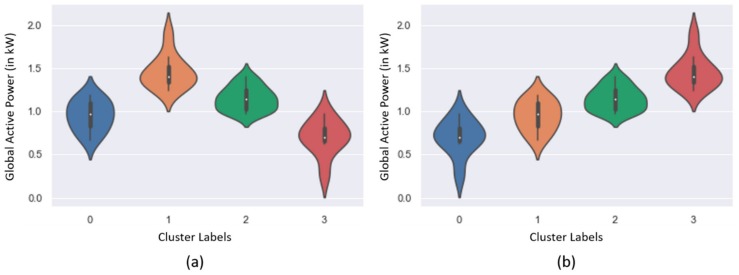
(**a**) The four clusters achieved by the proposed clustering technique, which were first randomly labelled with numbers ranging from 0 to 3, (**b**) The center of each cluster was identified and sorted in ascending order, which shows the proper numbers representing the level of consumption.

In order to achieve proper usage of the acquired clustering results, the energy consumption data was divided into various levels. Followed by this, appropriate levels of energy consumption were assigned to each cluster that were attained in the previous step. It has a bonus point where energy consumption analysts can easily differentiate among regions with a higher and lower level of energy consumption. When the dataset is divided into partitions, it is necessary to determine their degree of electricity energy utilization. For this purpose, the partitions were sorted according to their centers, and statistical measures were employed to identify which cluster represents a particular degree of electricity energy consumption. The median values of the features were chosen to compare the clusters. The reason why the median was chosen is that it is robust against outliers and skewed distributions. After computing the median of the feature, the clusters were sorted in ascending order according to their medians. In this manner, each cluster obtained its appropriate level of energy consumption. A sample representation for assigning levels to each cluster is given in Figure 4.

## 3. Experimental Results and Discussion

This section describes the experiments conducted to evaluate the proposed scheme on two benchmark energy consumption datasets including the DOE building performance [19] and individual household electric power consumption [20]. Various experiments were conducted using the raw features from datasets and encoded high dimensional features. Furthermore, the outcomes of clustering the energy consumption for yearly, monthly, weekly, and daily basis were discussed. The experimentation was carried out on R and Python 3.6 with RStudio and Jupyter Notebook IDEs on a Windows 10 OS with an Intel Core i7, 3.6 GHz processor, and 16 GB of RAM. In the experiments, the grid size was set to 2 × 2 and 4 × 1 while the temperature parameters $T_{max}\;$ and $T_{min}$ were 10.0 and 0.001, respectively. The training process was performed with a batch size of 5 and iterated for 10,000 times according to Algorithm 1.
**Algorithm 1** Clustering with SOM1: **Input:** Input data: *X*; SOM grid size *M, N*; Temperatures $T_{max},T_{min}$; Maximum iterations *MaxIter*;2: **Preparation:**3:     Pretrained deep autoencoder4: **Steps:**5:     **for** i **from** 0 **to**
*MaxIter*
**do**6:            Load training batch7:            Compute cluster assignments for the batch using Equation (3).8:            Update the temperature parameter using Equation (6).9:            Compute topographic weights for the batch using Equation (5).10:           Train autoencoder11:     **end for**12:           Assign energy consumption levels to clusters**Output:** Levels of energy consumption 

### 3.1. Evaluation Metrics

The evaluation of clustering algorithms is not a simple task compared to supervised classification algorithms. Generally, two approaches are used widely to judge the quality of the clustering algorithms: external and internal. External criteria are based on the knowledge about the ground truth class assignments of the data samples. However, that kind of prior information is usually not available or very costly to gather. Unlike the external evaluation method, internal criteria rely on information within the dataset. In this study, the three cluster validity indices were used to evaluate the performance of the proposed technique: The Silhouette coefficient [36], the Calinski–Harabasz [37], and the Davies–Bouldin indices [38].

The Silhouette score [36] judges the clustering performance based on the pairwise difference of inter- and intra-cluster distances. For a single sample in the dataset, the silhouette score is defined in Equation (7).

(7) $$s=\frac{b-a}{\min x\left(a,b\right)}\;$$

where *a* and *b* are the mean distances between one sample and all other points within the same partition and within the next nearest cluster, respectively. The overall score for the entire dataset is computed by taking the mean of all the silhouette scores of each point.

The Calinski–Harabasz (*CH*) index [37] is calculated by computing the ratio of the traces of intra- and inter-cluster scatter matrices for *K* clusters as given in Equation (8).

(8) $$CH=\left[\frac{\sum_{k=1}^{K}n_{k}\|z_{k}-z{\|}^{2}}{\Sigma_{k=1}^{K}\Sigma_{i=1}^{n_{k}}\|x_{i}-z_{k}{\|}^{2}}\right]\times \left[\frac{n-K}{K-1}\right]\;$$

where *n* is the number of samples in the dataset and $n_{k}$ is the number of points in cluster *k*. The *z* and $z_{k}$ are the centers of the entire data points and cluster *k*, respectively. Given the cluster diameters $s_{i}$ and $s_{j}$ of cluster i and j, and the distance between these two clusters $d_{ij}$, then the clustering algorithm is evaluated using Equation (9).

(9) $$R_{ij}=\frac{s_{i}+s_{j}}{d_{ij}}\;$$

This means that the lower the value of $R_{ij}$, the better the separation of the clusters and the compactness within the clusters. For instance, Davies–Bouldin (*DB*) index [38] for *k* clusters can be formulated as given in Equation (10).

(10) $$DB=\frac{1}{k}\overset{k}{\underset{i=1}{\sum }}\underset{i\neq j}{\max\;}R_{ij}$$

### 3.2. Results and Comparison with State-of-the-Art

The proposed clustering technique was compared with other conventional algorithms including k-means [31], ward [39], average-linkage [40], optics [33], birch [41], and fuzzy adaptive resonance theory (Fuzzy ART) [42], which is also based on competitive learning. The data for experiments over both datasets were split into training and test sets with a ratio of 4:1. The achieved results for the DOE building performance and individual household electricity power consumption datasets are given in Table 1 and Table 2, respectively. The scores are given in both tables where the highest score is represented in bold and the runner-up is underlined. The clustering validity indices with the dash sign indicate that a clustering algorithm yielded only a single cluster. The second last column shows whether or not the algorithm needs an input number of clusters and the retrieved clusters represent how many clusters have been generated by an algorithm for the given data.

Two kinds of experiments were performed for the proposed SOM clustering algorithm. First, the 2D-SOM structure with a grid size 2 × 2 was applied, however, the results achieved for this was not better than the k-means and others; it had the same accuracy as the state-of-the-art techniques. Next, the 1D-SOM structure with a grid size 4 × 1 was applied, and this architecture outperformed the state-of-the-art techniques. This can be explained by the fact that 1-dimensional SOM adjusts itself more easily to the underlying distribution of the dataset rather than 2-dimensional SOM [43]. The proposed method achieved the highest CH score of 690,916.65, and the second were Silhouette and DB scores of 0.58 and 0.49, respectively, for the DOE building performance dataset using the 1D-SOM architecture. The average linkage algorithm achieved the highest scores of 0.95 and 0.33 for Silhouette and DB, respectively. Similarly, the k-means achieved the second highest score of 683,580.737 for CH. Analogously, by using the individual household electricity power consumption dataset, the proposed method achieved the highest scores of 0.2412 and 85.2139 for Silhouette and CH, respectively, and was the second highest for DB. The average linkage achieved the highest for DB and second highest for Silhouette. Similarly, the k-means achieved the second highest for 84.1566 and 1.49 for CH and DB, respectively. The Birch and Fuzzy ART algorithms retrieved only one cluster for the DOE building performance dataset. Similarly, the Optics and Birch retrieved only one cluster for the individual household electricity power consumption dataset. The proposed clustering technique outperformed all of the clustering algorithms, except for the k-means and average-linkage hierarchical clustering algorithms in some cases. However, the advantage of 1D-SOM over k-means and average linkage is that, it does not enforce the number of clusters to be exactly equal to the input size. The SOM requires the maximum number of possible clusters and it adjusts very effectively to the number of clusters that can be generated from data. While assigning data to its units, some of the grid units may be empty with no sample assignment to it.

### 3.3. Cluster Visualizations and Analysis

**Figure 5:**
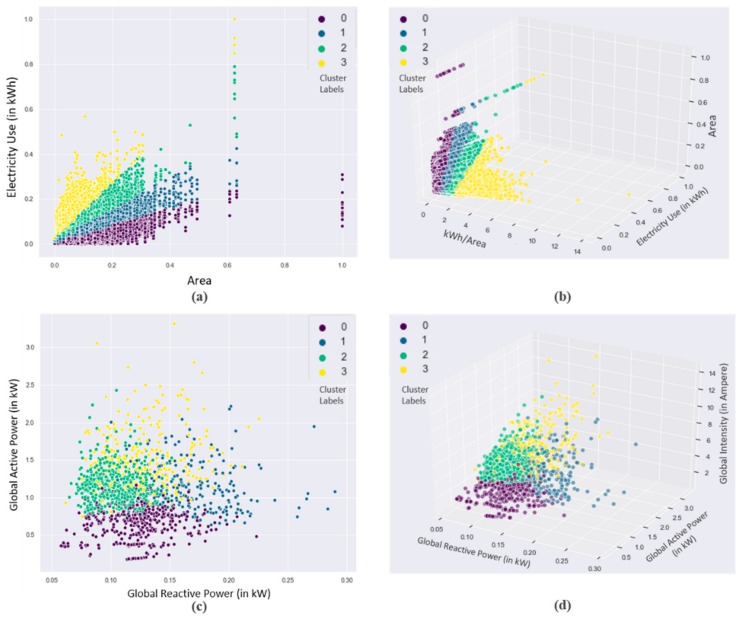
Clustering results of two datasets. (**a**,**b**) Residential DOE buildings dataset, (**c**,**d**) individual household dataset where each cluster is represented by a unique color based on different levels of consumption.

Visualization is a very important aspect of big data analytics. The clustering results have been visualized in various ways for the ease of energy providers. The residential DOE buildings dataset has the important feature of area, which is very helpful in knowing how much energy is consumed per area. The partitioning of this dataset is based on the rate of electricity energy consumption rate per area of the residential buildings. Therefore, Figure 5a,b show whether energy consumption is related to area or not.

**Figure 6:**
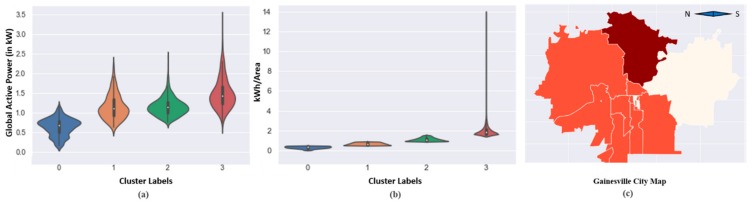
The distribution of samples among the levels of energy consumption. (**a**) Individual household electric power consumption, (**b**) DOE buildings performance dataset, and, (**c**) the map of Gainesville, where each area is a different color assigned based on the energy consumption of buildings in that area.

It is widely-known that an increase in building area will increase the energy consumption. However, using the proposed technique, the data are clustered in a way in which the higher consumption small area buildings are grouped with higher consumption large area buildings. This means that the area has some effect, but it cannot be said that the area is directly proportional to energy consumption; if the ratios of consumption are checked, they are similar. So, from this analysis, it can be derived as which buildings are consuming more from its daily needs. Furthermore, the levels of consumption were assigned to clusters for the residential DOE building dataset based on the median value of the electricity energy consumption rate per area in each cluster. For instance, in Figure 6b, cluster 0 had the smallest median value among other clusters, therefore it resembled the lowest level of energy consumption. Similarly, all clusters were assigned to their corresponding levels of energy usage from the lowest to highest. Afterward, the level of consumption was visualized on the map of Gainesville city. Since the coordinates of location of residential buildings were not available in the dataset, therefore, zip codes of the city were utilized, and the most frequent level of electricity utilization was selected for each area defined by zip codes; the results are presented in Figure 6c.

**Figure 7:**
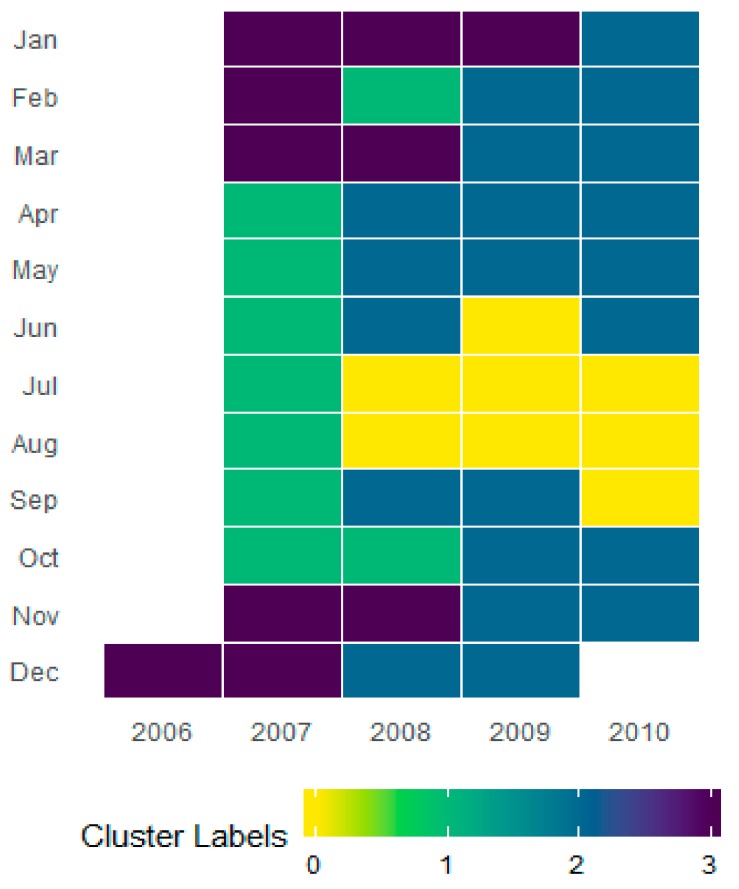
Monthly electricity energy consumption patterns captured in different clusters for the individual household dataset.

**Figure 8:**
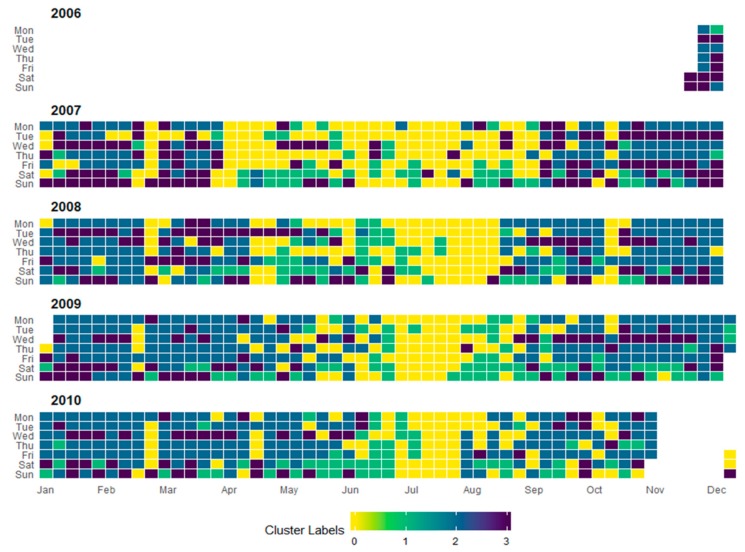
Daily electricity energy consumption patterns captured in different clusters during the five years for the individual household dataset.

For the individual household electricity power consumption, seven features were exploited for clustering and its analysis. Due to multiple features, the scattered plot visualization was not interpretable and ineffective in this case, as shown in Figure 5c,d. The 3D plot in Figure 5d was better for understanding the partition, however, it could not provide information about the feature dependency on the energy consumptions. Therefore, these data were analyzed on a monthly and daily basis and resampled for both cases in a different manner. The monthly consumption patterns provide broader details of energy consumption by a user and the daily levels prediction provide more detailed information about the individual usage of energy. For this dataset, the levels of consumption were identified based on the median value of Global Active Power Consumption and visualized in Figure 6a. Similarly, the levels of monthly consumption were predicted and illustrated in Figure 7. Finally, the daily profiles of the individuals are provided in Figure 8 for a period of five years and each level has been assigned a different color, which can easily indicate at what period of the year the user is consuming more energy.

## 4. Conclusions

In this paper, a clustering based electricity power consumption level prediction technique was presented for consumer profiling using smart sensor data. The proposed framework was based on a deep autoencoder and SOM. First, the smart sensor energy data were passed through a pre-processing step, where mean imputation and min-max methods are used for data normalization and outlier removal. Next, a deep autoencoder was trained to transfer the low dimensional energy data to high-level representations. These high-level representations were then fed into an adaptive SOM clustering algorithm. Finally, statistical analysis was done on the obtained clustered data to establish different levels of the electrical energy consumption level of each user. Furthermore, the energy consumption levels for efficient analysis were visualized by means of graphs and bar charts and we plotted the predicted levels on the map of a city, which clearly showed which part of the city had more energy consumption. Using this approach, providers can forecast each building’s energy consumption and produce that amount of energy in the future. Furthermore, it provides the timeline and behavior of energy utilization of each building’s energy utilization on a daily, weekly, monthly, and yearly basis. In future work, variational autoencoders can be utilized, which have the ability to understand the underlying probability distribution of the source data. Additionally, finding the parameters of the distribution can be investigated, which will help predict the variation in low dimensional data.

## Figures and Tables

**Table 1:** The comparison of the proposed technique with state-of-the-art clustering algorithms over DOE buildings performance dataset.

**Algorithm**	**Silhouette**	**CH**	**DB**	**Time (Seconds)**	**Input Clusters/** **Retrieved Clusters**	**Clusters Initialization** **Required/Not Required**
**k-means**	0.55	683,580.737	0.55	7.12	4/4	Required
**Ward**	0.48	580,575.728	0.55	1421.99	4/4	Required
**Average Linkage**	**0.95**	690.038	**0.33**	687.23	4/4	Required
**Optics**	0.17	27,847.348	59.03	10479.69	-/6	Not Required
**Birch**	-	-	-	4.57	-/1	Not Required
**Fuzzy ART**	-	-	-	13065.65	-/1	Not Required
**Proposed** **Method (1D SOM)**	0.58	**690916.65**	0.49	301.20	4/4	Required Maximum
**Proposed** **Method (2D SOM)**	0.55	661626.140	0.54	211.05	4/4	Required Maximum

**Table 2:** The comparison of the proposed technique with state-of-the-art clustering algorithms over the individual household electric power consumption dataset.

**Algorithm**	**Silhouette**	**CH**	**DB**	**Time (Seconds)**	**Input Clusters/** **Retrieved Clusters**	**Cluster Initialization** **Required/Not Required**
**k-means**	0.2011	84.1566	1.49	0.13	4/4	Required
**Ward**	0.1643	335.12	1.63	0.15	4/4	Required
**Average Linkage**	0.227	5.27	**0.44**	0.18	4/4	Required
**Optics**	-	-	-	1.21	-/1	Not Required
**Birch**	-	-	-	0.025	-/1	Not Required
**Fuzzy ART**	0.06	83.987	3.530	89.91	-/14	Not Required
**Proposed** **Method (1D SOM)**	**0.2412**	**85.2139**	1.49	241.05	4/4	Required Maximum
**Proposed** **Method (2D SOM)**	0. 1994	60.5528	2.21	274.64	4/4	Required Maximum
